# Dr. Edward Rigby

**Published:** 1861-03

**Authors:** 


DR. EDWARD RIGBY expired in London, on the twenty-seventh of December, in the fifty-sixth year of his age. The immediate cause of death was congestion of the lungs and suppression of the renal secretion; but he was also affected with cancer of the bladder, and for the last few days his sufferings were excruciating. In 1825, at the age of twenty-one, Dr. Rigby, although the medical profession was not his choice, took his degree of Doctor of Medicine, at the University of Edinburgh; and then went to Berlin, where he spent one year in studying midwifery. From this city he repaired to Heidelberg, and at the Lying-in Hospital made himself conversant with the mechanism of labor. On returning to England in 1829, he became house pupil at the Westminster Lying-in Hospital, and translated Nægelé's essay "On the Mechanism of Parturition." In 1831 he was appointed assistant lecturer on Midwifery, at St. Thomas's Hospital, and afterward succeeded Dr. Gooch as physician to the Lying-in Hospital, an appointment which he held until within two years of his death. In 1837 he was made lecturer on Midwifery at St. Bartholomew's Hospital, which he held fourteen years; and at the time of his death he was President of the Obstetrical Society of London.

Dr. Rigby was well known as a clear and forcible writer. In 1843 he published a second edition of Hunter's "Anatomical Description of the Human Gravid Uterus," and a year later a work entitled "On Dysmenorrhœa and other Uterine Affections in Connection with Derangement of the Assimilating Functions." In the same year appeared his "System of Midwifery," which for some time occupied the first position in England as a textbook on Midwifery; and in 1857 he published a work "On the Constitutional Treatment of Female Diseases."

